# Combined Aerobic and Resistance Exercise Training Improve Hypertension Associated With Menopause

**DOI:** 10.3389/fphys.2018.01471

**Published:** 2018-10-29

**Authors:** Guilherme Lemos Shimojo, Danielle da Silva Dias, Christiane Malfitano, Iris Callado Sanches, Susana Llesuy, Luis Ulloa, Maria-Cláudia Irigoyen, Kátia De Angelis

**Affiliations:** ^1^Laboratory of Translational Physiology, Universidade Nove de Julho (UNINOVE), São Paulo, Brazil; ^2^Human Movement Laboratory, São Judas Tadeu University, São Paulo, Brazil; ^3^Departamento de Química Analítica y Fisicoquímica, Facultad de Farmacia y Bioquímica, Universidad de Buenos Aires, Buenos Aires, Argentina; ^4^Center for Immunology and Inflammation, Department of Surgery, Rutgers-New Jersey Medical School, Rutgers University, Newark, NJ, United States; ^5^Heart Institute, University of São Paulo Medical School, São Paulo, Brazil; ^6^Department of Physiology, Federal University of São Paulo, São Paulo, Brazil

**Keywords:** combined exercise training, ovariectomy, hypertension, cardiovascular autonomic dysfunction, oxidative stress, inflammation, kidney

## Abstract

The prevalence of hypertension sharply increases in menopausal women. Recent studies have demonstrated that aerobic or resistance training may help control hypertension. In this study, we report that combining aerobic and resistance training may provide an effective therapeutic approach for hypertension control, attenuating inflammation and oxidative stress in ovariectomized rats. Female Wistar and spontaneous hypertensive rats (SHR) were distributed into four groups: sedentary control (C), sedentary hypertensive (HR), sedentary hypertensive ovariectomized (HR-O), and combined trained hypertensive ovariectomized (T-HR-O). Combined exercise training was performed on a motor treadmill (aerobic training) and on a ladder adapted to rats (resistance training), in alternate days for 8 weeks. Direct arterial pressure was recorded and oxidative stress and inflammation were evaluated in cardiac and renal tissue. Ovariectomy increases increased mean arterial blood pressure, sympathetic modulation, and oxidative stress in SHR. Combining aerobic and resistance training reduced mean arterial blood pressure (12% vs. HR-O), heart rate (8% vs. HR-O), vascular sympathetic modulation (40% vs. HR-O), and improved baroreflex sensitivity. Combined training reduced cardiac inflammation (TNF and IL-6) and cardiac and renal lipoperoxidation (59% and 57%, respectively vs. HR-O). It also enhanced cardiac (71%) and renal (76%) total antioxidant capacity when compared to HR-O group. In conclusion, combining aerobic and resistance training improves mean arterial blood pressure, cardiovascular autonomic control, preventing cardiac and renal oxidative stress and inflammation in an experimental hypertension model with surgical menopause induced with ovariectomy.

## Introduction

Hypertension is one of the main factors contributing to cardiovascular disorders (Mozaffarian et al., 2016). Epidemiological studies have shown that menopausal women have a high prevalence of hypertension, regardless of their ethnicity (Cutler et al., 2008). Although the mechanisms underlying this increased susceptibility remain unclear, recent studies have suggested that menopausal women have increased sympathetic tone, which in turn may increase arterial blood pressure and lead to renal damage (Hogarth et al., 2011; Maranon et al., 2014). Both chronic inflammation and oxidative stress contribute to the pathogenesis of hypertension (Watson et al., 2008; Gocmen et al., 2014). The immune system releases cytokines, which cause oxidation (Kaur et al., 2006; Crowley, 2014). In turn, oxidative stress induces local inflammation by increasing endothelial permeability and sustaining inflammation (Bjorkbacka, 2006; Sima et al., 2009). Oxidative stress contributes to the pathophysiology of a range of diseases, such as cancer, hypertension, cardiomyopathy, cardiac hypertrophy, and congestive heart failure (Valko et al., 2004; Chatterjee et al., 2007; Ceriello, 2008; Pham-Huy et al., 2008). It is important to highlight that studies have observed that autonomic nervous system can modulate inflammation, and consequently, oxidative stress (Pavlov and Tracey, 2012). In this sense, a recent study from our group reported that an impairment in baroreflex sensitivity, which is an excellent index of autonomic dysfunction, preceded changes in markers of inflammation and oxidative stress in hypertensive rats submitted to fructose overload (Bernardes et al., 2018). Taken together, these findings suggest that sympathetic dysfunction can be the trigger to hypertension development through increased inflammation and oxidative stress in menopausal women.

Several studies have indicated that exercise is an important non-pharmacological strategy to prevent and treat hypertension (Pescatello et al., 2004; Cornelissen and Smart, 2013). Aerobic exercise decreases sympathetic tone, arterial blood pressure, oxidative stress (Irigoyen et al., 2005; Bertagnolli et al., 2006), and inflammation (Pan et al., 2007; Masson et al., 2014).

On the other hand, resistance training may also induce positive effects on several menopause-related diseases (Brochu et al., 2009; Zaki, 2014). Thus, we reasoned that the combination of aerobic and resistance training would maximize musculoskeletal and cardiovascular therapeutic benefits for menopausal women (Williams et al., 2007). Despite the large number of studies focusing on either aerobic or resistance training, it remains unclear whether combining these two exercises can promote beneficial effects in menopausal women to control hypertension.

We hypothesize that surgical ovariectomy causes ovarian hormone deprivation, as observed in menopausal women, and further increases susceptibility to hypertension, cardiovascular autonomic dysfunction, inflammation, and oxidative stress in hypertensive rats (SHR). Given the European Society of Cardiology and American College of Sports Medicine guidelines’ recommending exercise for the management of hypertension, we also hypothesize that the combination of aerobic and resistance training would prevent autonomic and cardiovascular dysfunction in these animals. Therefore, we assessed the effects of combined exercise on hemodynamic parameters, cardiovascular autonomic dysfunction, inflammation, and oxidative stress in hypertensive ovariectomized rats.

## Materials and Methods

### Animal Experimentation

Female normotensive Wistar rats (WKY, *n* = 7) and spontaneous hypertensive rats (SHR, *n* = 21) (3 months) were obtained from the Animal Facility of Universidade Nove de Julho. The animals were freely fed with standard laboratory chow (Nuvilab, Brazil) and water and were housed in temperature-controlled rooms (22°C) with a 12: 12-h dark–light cycle. The rats were assigned into four groups (*n* = 7 each): sedentary control (C), sedentary hypertensive (HR), sedentary hypertensive ovariectomized (HR-O), and combined trained hypertensive ovariectomized (T-HR-O) groups. All surgical procedures and protocols were approved by the Ethics Committee of Universidade Nove de Julho (An0019/2013) and were conducted in accordance with the National Institutes of Health Guide for the Care and Use of Laboratory Animals.

### Combined Exercise Training

Combined exercise training was performed on a motor treadmill (aerobic training) and on a ladder adapted for rats (resistance training), on alternate days, 5 days/week, for 8 weeks. For accuracy of prescription, maximal (running or load) tests were performed at the beginning of the experiment and in the 4th and 8th weeks of the training protocol.

### Ovariectomy

In the third month of life, the animals were anesthetized (80 mg/kg ketamine and 12 mg/kg xylazine, intraperitoneal, i.p), and a small abdominal incision was performed. The oviduct was sectioned and the ovary removed.

### Aerobic Exercise Training

All animals were adapted to walk and run on a motorized treadmill (10 min/day; 0.3 km/h) for 5 consecutive days before the maximal running test. Sedentary and trained rats underwent the maximal running test in the first week following ovariectomy, as described in detail in a previous study (Rodrigues et al., 2007). Aerobic exercise training was performed on a treadmill (Imbramed TK-01, Brazil) at low-to-moderate intensity (∼50–60% maximal running speed) for 1 h a day, 5 days a week for 8 weeks. In order to provide similar environment and manipulation, sedentary animals were placed on the stationary treadmill three times a week.

### Resistance Exercise Training

Based on our previous experience with aerobic exercise training using a treadmill, the animals were gradually adapted to the act of climbing for 5 consecutive days before the maximal load test. This is a voluntary exercise protocol, without aversive (electrical) stimulation to maintain performance, restraint, and use of food or water as motivators. The dynamic resistance exercise test consisted of an initial load of 75% of body weight. After a 2-min resting period, an additional 15% of body weight was used in the subsequent climbs, as previously detailed elsewhere (Sanches et al., 2014). The protocol of resistance exercise training was performed using the normalized value of maximal load for each rat, and was adjusted weekly, according to the body weight of the animal. The resistance exercise training protocol was performed during 8 weeks, for 5 days a week, and at moderate intensity (1st–2nd week: 30–40%; 3rd–5th week: 40–50%; and 6th–8th week: 40–60% of the maximal load) with 15 climbs per session and a 1-min time interval between climbs, as previously detailed elsewhere (Sanches et al., 2014). Importantly, to maintain the standard of 6 climbs for maximal load, load increment was adjusted to the maximal load test performed at weeks 4 and 8 of the protocol, with +25% and +40% of the body weight increments between climbs, respectively. The purpose of such test was to determine both physical capacity and exercise training intensity.

### Cardiovascular Measurements

On the day following the last exercise session, rats were anesthetized with an intraperitoneal injection of ketamine (90 mg/kg) and xylazine (20 mg/kg) to implant 2 polyethylene-tipped Tygon cannulas filled with heparinized saline into the right carotid artery and jugular vein for direct measurements of arterial blood pressure (AP) and drug administration, respectively. Cannulated rats were treated with antibiotic and analgesics and were allowed one day of recovery. All efforts were made to avoid suffering. Throughout the experiment, rats received food and water *ad libitum*; they remained conscious in their cages and were allowed to move freely during hemodynamic measurements. To avoid detraining, hemodynamic measurements were made in conscious, freely moving rats in their home cage 24 h after surgery (16, 37). The arterial cannula was connected to a transducer (Blood Pressure XDCR, Kent^®^ Scientific, United States), and AP signals were recorded for a 30-min period using a microcomputer equipped with an analog-to-digital converter (CODAS, 2Kz, DATAQ Instruments, United States). The recorded data were analyzed on a beat-to-beat basis to quantify changes in systolic (SAP), diastolic (DAP), and mean AP (MAP) and heart rate (HR).

### Cardiovascular Autonomic Measurements

After basal AP measurements, baroreflex sensitivity was evaluated using increasing doses of phenylephrine (0.5–2.0 μg/mL) and sodium nitroprusside (5–20 μg/mL), given as sequential bolus injections (0.1 mL) to produce AP rise and fall responses ranging from 5 to 40 mmHg each. Baroreflex sensitivity was assessed by a mean index relating changes in HR to changes in MAP, allowing a separate analysis of gain for reflex bradycardia and reflex tachycardia as described elsewhere (Flues et al., 2010; Sanches et al., 2012; Bernardes et al., 2018). SD from the mean of three time series of 5 min for each animal was used to obtain the pulse interval (PI) and SAP variabilities in time-domain. For frequency domain analysis, the same time series of PI and SAP were cubic spline interpolated (250 Hz) and cubic spline decimated to be equally spaced in time after linear trend removal; power spectral density was obtained through the Fast Fourier Transformation. Spectral power for low-frequency (LF; 0.20–0.75 Hz) and high-frequency (HF; 0.75–4.0 Hz) bands were calculated by power spectrum density integration within each frequency bandwidth, using a customized routine (MATLAB 6.0, Mathworks). The coherence between the PI and SAP signal variability was assessed through cross-spectral analysis (Sanches et al., 2012).

### Tissue Preparation

After cardiovascular measurements, the animals were pre-anesthetized with ketamine and killed by decapitation, the heart (ventricles) and kidney (right) were immediately removed, rinsed in saline, and trimmed to remove fat tissue and visible connective tissue.

### Inflammatory Mediators

Interleukin 6 (IL-6), IL-10, and tumor necrosis factor alpha levels were determined in cardiac and renal tissues using a commercially available ELISA kit (R&D Systems Inc.), in accordance with the manufacturer’s instructions. ELISA was performed in 96-well polystyrene microplate with a specific monoclonal antibody coating. Absorbance was measured at 540 nm in a microplate reader.

### Oxidative Stress Evaluations

Cardiac and renal tissues were then cut into small pieces, placed in ice-cold buffer, and homogenized in an ultra-Turrax blender with 1 g of tissue per 5 mL of 120 mmol/L KCl and 30 nmol/L phosphate buffer, pH 7.4. Homogenates were centrifuged at 600 g for 10 min at 4°C. Protein was determined as described previously (Lowry et al., 1951).

### Lipoperoxidation-Thiobarbituric Acid Reactive Substances (TBARS)

For the TBARS assay, trichloroacetic acid (10%, w/v) was added to the homogenate to precipitate proteins and to acidify the samples. This mixture was then centrifuged (3,000 rpm, 3 min), the protein-free sample was extracted, and thiobarbituric acid (0.67%, w/v) was added to the reaction medium. The tubes were placed in a water bath (100°C) for 15 min. Absorbance was measured at 535 nm using a spectrophotometer. A commercially available malondialdehyde was used as a standard, and the results are expressed as nanomoles per milligram of protein (Ohkawa et al., 1979).

### Protein Oxidation

This method uses the reaction of protein carbonyl groups with 2,4-dinitrofenylhydrazyne to form a 2,4-dinitrophenylhydrazone, which can then be measured spectrophotometrically at 360 nm, as previously described (Reznick and Packer, 1994).

### Total Radical-Trapping Antioxidant Potential (TRAP)

TRAP, which indicates the total antioxidant capacity present in a homogenate was measured by chemiluminescence using 2,2′-azo-bis(2-amidinopropane) (ABAP, a source of alkyl peroxyl free radicals) and luminal. A mixture consisting of 20 mmol.L-1luminol and 50 mmol.L^−1^ phosphate buffer (pH = 7.4) was incubated to achieve a steady-state luminescence from the free radical-mediated luminal oxidation. A calibration curve was obtained by using different concentrations (between 0.2 and 1 μmol.L^−1^) of Trolox (hydrosoluble vitamim E). Luminescence was measured in a liquid scintillation counter using the out-of-coincidence mode (Evelson et al., 2001).

### Antioxidant Enzymes

Superoxide dismutase activity was measured spectrophotometrically by the rate inhibition of pyrogallol auto-oxidation at 420 nm. (26) Enzyme activity was reported as U/mg protein. CAT concentration was measured by monitoring the decrease in H_2_O_2_ concentration at 240 nm, and the results are reported as pmol of H_2_O_2_/mg protein (Aebi, 1984).

### NADPH Oxidase

The nicotinamide adenine dinucleotide phosphate oxidase (NADPH oxidase) was determined by ELISA (SEA554Ra, USCN Life Science, United States) in renal tissue.

### Statistical Analysis

All tests were performed using the GraphPad Prism Software^®^ (GraphPad Software, La Jolla, CA, United States). Data are presented as mean ± SEM. Levene’s test was used to assess variance homogeneity. Comparisons between the four groups were performed with one-way ANOVA, followed by Student Newmann Keuls *post hoc* test. The significance level was established at *p* < 0.05.

## Results

### Body Weight

We first noticed that 3-month-old female normotensive Wistar (WKY) control rats had higher body weight than their counterpart SHR (C: 216 ± 5 g vs. HR: 188 ± 2 g; *p* < 0.05). All hypertensive rats had statistically similar body weight, regardless of their protocol (HR: 188 ± 2 g; HR-O: 191 ± 2; and T-HR-O: 191 ± 2 g, *p* < 0.05). During the 8 weeks of the experimental procedure, normotensive animals presented a significant 25% body weight increase (initial = 216 g vs. final = 280 g). Hypertensive animals without treatment presented a slight 5% body weight increase, but ovariectomy increased by nearly 38% of the body weight of hypertensive rats. Exercise did not affect this body weight gain and these animals gained 33% in body weight during the 8-week protocol (Table 1).

**Table 1:** Body weight in the groups C, sedentary control (*n* = 7); HR, sedentary hypertensive (*n* = 7); HR-O, sedentary hypertensive ovariectomized (*n* = 7); and T-HR-O, combined trained hypertensive ovariectomized (*n* = 7).

Measurement	C	HR	HR-O	T-HR-O
Initial				
Weight (g)	216 ± 5	188 ± 2^†^	191 ± 2^†^	191 ± 2^†^
Final				
Weight (g)	280 ± 5	197 ± 2^†^	264 ± 2^†∗^	255 ± 2^∗#†^
Gain				
Weight (g)	64 ± 2	9 ± 1^†^	73 ± 2^†^	64 ± 3^∗#^
% gain	29%	5%	38%	33%

*Data are reported as mean ± SEM. Gain weight: final body weight–initial body weight. % gain: (gain weight/initial body weight)^∗^100. ^†^p < 0.05 vs. C; ^∗^p < 0.05 vs. HR; ^#^p < 0.05 vs. HR-O. MAP, mean arterial pressure; SAP, systolic arterial pressure; DAP, diastolic arterial pressure; HR, heart rate.*

### Maximal Exercise Capacity

We used two tests to determine the exercise capacity of the animals: maximal speed in the treadmill and the ladder load test. Control normotensive animals have a lower exercise capacity than any of the hypertensive animal groups in both maximal speed and load resistance (1.8 ± 0.7 km/h, *p* < 0.05 and 128 ± 5.9 g, *p* < 0.05, respectively). All hypertensive animals presented a higher exercise capacity than the control animals. However, all hypertensive animal groups had similar exercise capacity, regardless of ovariectomy before training (HR: 2.4 ± 0.6; HR-O: 2.5 ± 0.6 and T-HR-O: 2.4 ± 0.4 km/h, *p* > 0.05) (HR: 315 ± 18.7; HR-O: 320 ± 12.6 and T-HR-O: 329 ± 6.8 g, *p* > 0.05). After the 8 weeks experimental protocol, all untrained animal groups exercise maintained their exercise capacity. Only the exercise trained animals increased their capacity both in the maximal speed (T-HR-O: 3.4 ± 0.5 vs. C: 1.8 ± 1.5; HR: 2.4 ± 0.6 and HR-O: 2.3 ± 0.6 km/h, *p* < 0.05) and load capacity (T-HR-O: 500 ± 9.7 vs. C: 204 ± 10.8; HR: 360 ± 19.5 and HR-O: 335 ± 23.8 g, *p* < 0.05).

### Cardiovascular Analyses

The hemodynamic cardiovascular assessment indicated that ovariectomy increased the mean arterial blood pressure (HR-O: 176 ± 3.7 vs. HR: 165 ± 3.8 mmHg) and combined training prevented the effect of ovariectomy and lowered hypertension (Table 2). Likewise, all hypertensive animal groups had higher diastolic and systolic arterial pressure and ovariectomy further increased both parameters. Again, combined training seemed to have prevented the effect of ovariectomy and lowered both the diastolic and systolic arterial pressure. Heart rate was similar among all normo and hypertensive animals with the exception of the trained animals. Combined training decreased heart rate by 8% in hypertensive ovariectomized animals.

**Table 2:** Hemodynamic and cardiac autonomic control in C, sedentary control (*n* = 7); HR, sedentary hypertensive (*n* = 7); HR-O, sedentary hypertensive ovariectomized (*n* = 7); and T-HR-O, combined trained hypertensive ovariectomized (*n* = 7).

Measurement	C	HR	HR-O	T-HR-O
MAP (mmHg)	113 ± 1.5	165 ± 3^†^	176 ± 4^†∗^	155 ± 3^†#∗^
DAP (mmHg)	95 ± 2	145 ± 3^†^	154 ± 3.4^†∗^	133 ± 3^†#∗^
SAP (mmHg)	128 ± 2	192 ± 4^†^	200 ± 5^†^	177 ± 4^†#∗^
HR (bpm)	366 ± 11	359 ± 7	355 ± 6	330 ± 6^†#∗^

*Data are reported as mean ± SEM. ^†^p < 0.05 vs. C; ^∗^p < 0.05 vs. HR; ^#^p < 0.05 vs. HR-O. MAP, mean arterial pressure; DAP, diastolic arterial pressure; SAP, systolic arterial pressure; HR, heart rate.*

### Assessment of Cardiovascular Autonomic Parameters

**FIGURE 1:**
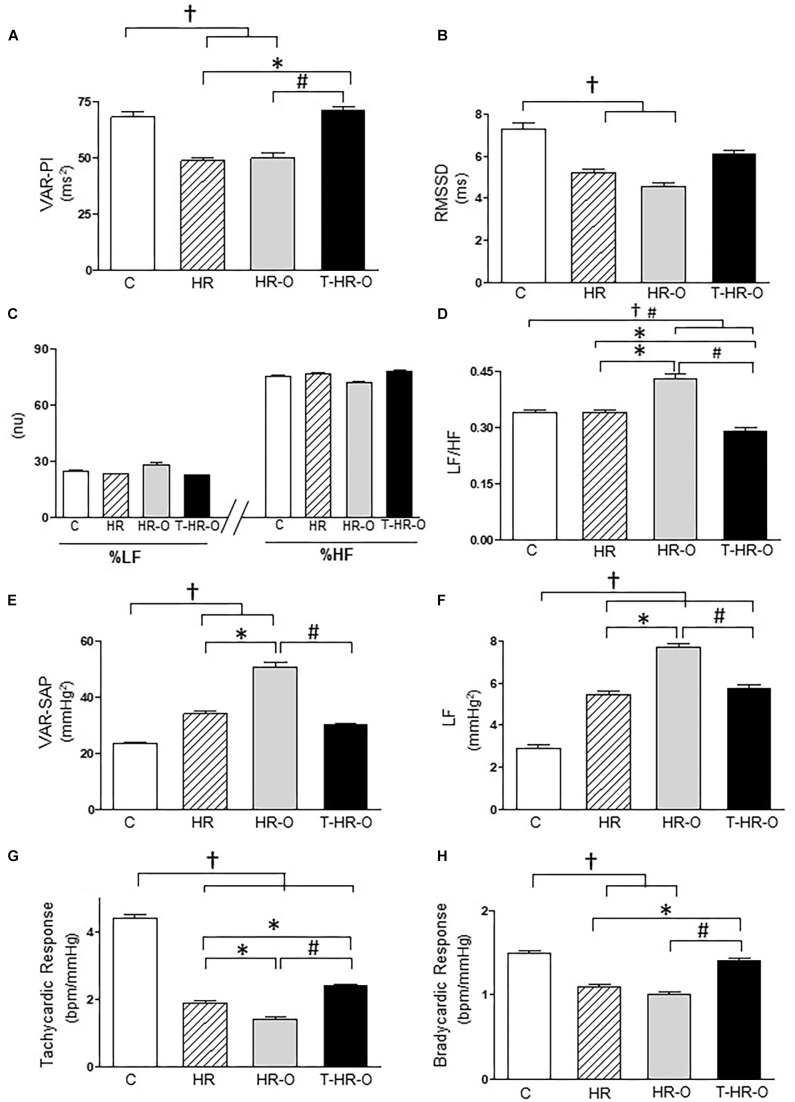
**(A)** Pulse interval variability (VAR-PI). **(B)** Root mean square of successive differences in the pulse interval (RMMSD). **(C)** Percentage of LF (low-frequency band, 0.20–0.75 Hz) and HF (high-frequency band, 0.75–3 Hz) band. **(D)** LF/HF ratio. **(E)** Total variance of systolic arterial pressure (VAR-SAP). **(F)** Low-frequency band of SAP (LF-SAP). **(G)** Baroreflex evaluated by bradycardic and **(H)** tachycardic responses. **(C)** sedentary control (*n* = 7); HR: sedentary hypertensive (*n* = 7); HR-O: sedentary hypertensive ovariectomized (*n* = 7); and T-HR-O: combined trained hypertensive ovariectomized (*n* = 7). ^†^*P* < 0.05 vs. C; ^∗^*P* < 0.05 vs. HR; ^#^*P* < 0.05 vs. HR-O.

The hypertensive animal groups presented lower total variance of pulse interval (VAR-PI) (HR: 48.66 ± 3.3 and HR-O: 49.77 ± 6.7 ms^2^) and root mean square of successive differences in the pulse interval (RMSSD) (HR: 48.66 ± 3.3 and HR-O: 49.77 ± 6.7 ms^2^, HR: 5.23 ± 0.4 and HR-O: 4.56 ± 0.5 ms, respectively). Combined training increased VAR-PI (71.04 ± 4.5 ms^2^) and RMSSD (6.06 ± 0.6 ms) (Figures 1A,B). All animal groups had statistically similar low- and high-frequency autonomic activity, regardless of ovariectomy and the exercise training (Figure 1C). Exercise training decreased sympathetic modulation, and trained animals showed lower LF/HF ratio (T-HR-O: 0.29 ± 0.03 vs. C: 0.34 ± 0.02; HR: 0.34 ± 0.02; and HR-O: 0.43 ± 0.04, *p* < 0.05) (Figure 1D). Again, exercise training reduced the variability and the low-frequency band of systolic arterial pressure, suggesting a decrease in sympathetic autonomic modulation (T-HR-O: 30.09 ± 2.0 vs. HR: 34.09 ± 2.4 and HR-O: 50.78 ± 4.6 mmHg^2^, T-HR-O 5.72 ± 0.6 vs. HR-O: 7.69 ± 0.5 mmHg^2^, *p* < 0.05, respectively) (Figures 1E,F). We also determined baroreflex sensitivity by analyzing the tachycardic and bradycardic responses to phenylephrine or sodium nitroprusside, respectively. Hypertensive animals presented lower tachycardic (HR: 1.9 ± 0.2 and HR-O: 1.4 ± 0.2 vs. C: 4.4 ± 0.3 bpm/mmHg, *p* < 0.05) and bradycardic responses (HR: −1.1 ± 0.08 and HR-O: −1.0 ± 0.09 vs. C: −1.5 ± 0.08 bpm/mmHg, *p* < 0.05). Exercise increased both the tachycardic (T-HR-O: 2.4 ± 0.09 bpm/mmHg, *p* < 0.05) and the bradycardic responses (T-HR-O: −1.3 ± 0.09 bpm/mmHg, *p* < 0.05) to phenylephrine or sodium nitroprusside, respectively (Figures 1G,H).

### Immune Evaluation

We evaluated the effect of exercise on the inflammation associated with hypertension. We analyzed inflammatory factors in the main organs controlling cardiovascular hemodynamics, including the heart and kidneys (Table 3). All hypertensive animal groups showed higher TNF cardiac and kidney levels than normotensive animals. Ovariectomy elevated renal TNF, but not cardiac levels. By contrast, exercise decreased cardiac TNF, but not kidney levels. On the other hand, all hypertensive animal groups had lower IL6 cardiac levels, regardless of ovariectomy. As in the case of TNF, exercise training also reduced IL6 cardiac levels, but it did not lower IL6 in the kidneys. We also analyzed the anti-inflammatory cytokine IL10. Normotensive and hypertensive animals had similar IL10 cardiac and kidney levels. Ovariectomy decreased both IL10 cardiac levels and kidney levels. By contrast, exercise training increased renal IL10, but not cardiac levels. Thus, exercise training lowers inflammatory cytokines (TNF and IL6) in the heart, but not in the kidneys, and increases anti-inflammatory cytokine IL10 in the kidneys, but not in the heart.

**Table 3:** Inflammatory response in cardiac tissue in C, sedentary control (*n* = 7); HR, sedentary hypertensive (*n* = 7); HR-O, sedentary hypertensive ovariectomized (*n* = 7); and T-HR-O, combined trained hypertensive ovariectomized (*n* = 7).

Measurement	C	HR	HR-O	T-HR-O
Cardiac				
TNF (pg/mg protein)	36.05 ± 5.1	61.71 ± 7.2^†^	60.72 ± 8.4^†^	49.5 ± 4.0
IL6 (pg/mg protein)	200 ± 21.15	156 ± 26.03	155 ± 20.68	115 ± 6.47^†^
IL10 (pg/mg protein)	59.05 ± 7.05	54.19 ± 10.23	33.16 ± 5.98^†^	29.45 ± 4.35^†^
Renal				
TNF (pg/mg protein)	81.24 ± 8.87	88.93 ± 4.75	96.34 ± 3.70	91.33 ± 7.23
IL6 (pg/mg protein)	139 ± 19.20	144 ± 16.79	121 ± 15.19	126 ± 12.36
IL10 (pg/mg protein)	45.71 ± 3.03	54.33 ± 3.49	43.44 ± 3.81	60.08 ± 4.35^#^

*Data are reported as mean ± SEM. ^†^p < 0.05 vs. C; ^#^p < 0.05 vs. HR-O. TNF, tumor necrosis factor; IL6, interleukin 6; IL10, interleukin 10.*

### Oxidative Stress

**FIGURE 2:**
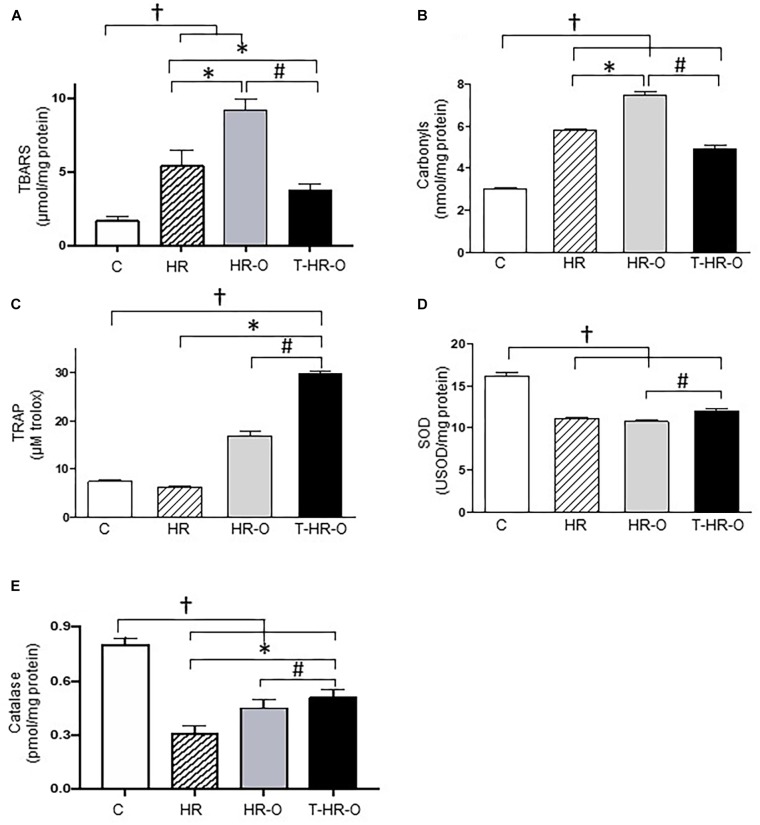
Cardiac oxidative stress assessed by **(A)** lipoperoxidation (TBARS), **(B)** carbonyls, **(C)** total radical-trapping antioxidant parameter (TRAP), **(D)** superoxide dismutase (SOD), and **(E)** catalase. C: sedentary control (*n* = 7); HR: sedentary hypertensive (*n* = 7); HR-O: sedentary hypertensive ovariectomized (*n* = 7); and T-HR-O: combined trained hypertensive ovariectomized (*n* = 7). ^†^*P* < 0.05 vs. C; ^∗^*P* < 0.05 vs. HR; ^#^*P* < 0.05 vs. HR-O.

**FIGURE 3:**
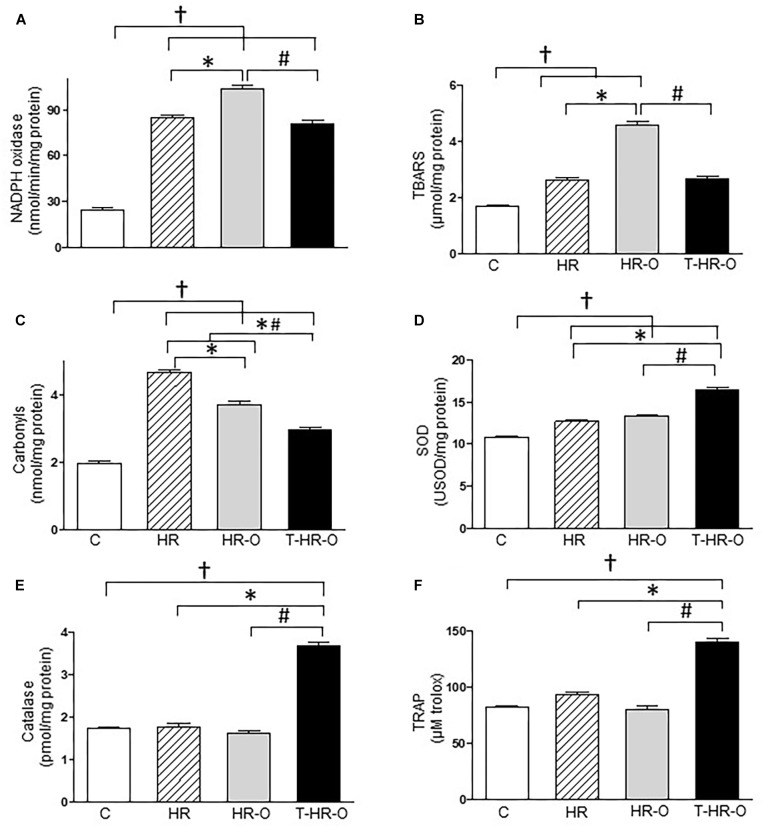
Kidney oxidative stress assessed by **(A)** NADPH oxidase, **(B)** lipoperoxidation (TBARS), **(C)** carbonyls, **(D)** superoxide dismutase (SOD), **(E)** catalase, and **(F)** total radical-trapping antioxidant parameter (TRAP). C: sedentary control (*n* = 7); HR: sedentary hypertensive (*n* = 7); HR-O: sedentary hypertensive ovariectomized (*n* = 7); and T-HR-O: combined trained hypertensive ovariectomized (*n* = 7). ^†^*P* < 0.05 vs. C; ^∗^*P* < 0.05 vs. HR; ^#^*P* < 0.05 vs. HR-O.

Hypertensive animals presented higher cardiac lipoperoxidation (HR: 5.4 ± 1.1 vs. C: 1.7 ± 0.3 μmol/mg protein, *p* < 0.05). Ovariectomy significantly enhances both cardiac lipoperoxidation and protein oxidation (HR-O: 9.2 ± 0.8 μmol/mg protein, HR-O: 3.0 ± 0.2 nmol/mg protein). Conversely, exercise training reduced both cardiac lipoperoxidation and protein oxidation (T-HR-O: 3.8 ± 0.4 μmol/mg protein, T-HR-O: 5.8 ± 0.3 nmol/mg protein) (Figures 2A,B). Ovariectomy also increased TRAP in the cardiac tissue, and exercise training further increased these values (T-HR-O: 29.8 ± 1.2 vs. C: 7.4 ± 1.2; HR: 6.2 ± 0.5 and HR-O: 16.8 ± 2.6 μM Trolox, *p* < 0.05) (Figure 2C). All hypertensive animals groups showed lower cardiac SOD activity, and exercise training increased both cardiac SOD and CAT activities (Figures 2D,E). Ovariectomy significantly increased renal NADPH oxidase activity, while exercise training lowered this marker of oxidative stress (T-HR-O: 80.8 ± 7.1 vs. HR-O: 103.6 ± 5.6 ng/ml, *p* < 0.05) (Figure 3A). Hypertensive animals presented higher renal lipoperoxidation and protein oxidation, and both were increased by ovariectomy. Again, exercise training decreased both renal lipoperoxidation and protein oxidation, while increasing renal SOD, CAT, and TRAP activity in hypertensive ovariectomized animals (Figures 3B–F).

## Discussion

Given that exercise improves autonomic dysfunction, and that this dysfunction contributes to hypertension, we assessed whether regular exercise controls hypertension in an experimental model of menopausal women. Previous clinical and experimental studies have focused on the effects of either aerobic (Irigoyen et al., 2005; Bertagnolli et al., 2006; Pan et al., 2007; Masson et al., 2014) or resistance training on several menopause-related disorders (Brochu et al., 2009; Zaki, 2014; Shimojo et al., 2015; da Palma et al., 2016). Despite the large number of studies looking at the benefits of either aerobic or resistance exercise, it was still unclear whether the combination of these two exercises modalities would promote further beneficial effects on hypertension control in menopausal women. Our findings provide three major insights. First, combining exercise reduces AP and HR at rest. Second, it promotes beneficial adaptation in baroreflex sensitivity and cardiovascular autonomic modulation. Third, these improvements in baroreflex sensitivity and cardiovascular autonomic modulation may mediate the potential of exercise to decrease cardiac and renal inflammation and oxidative stress in hypertensive ovariectomized animals.

Baroreflex sensitivity and heart rate variability are significant predictors of cardiac mortality (La Rovere et al., 2002) and these have been reported in experimental and clinical trials of hypertension (Bristow et al., 1969; Bertagnolli et al., 2006). Our results show that hypertensive animals with or without ovariectomy had decreased baroreflex sensitivity associated with increased AP levels. Our results concur with previous studies associating baroreflex sensitivity with increased AP in male SHR (Bertagnolli et al., 2006; Masson et al., 2014). Combined exercise also improves tachycardic and bradycardic responses to sodium nitroprusside and phenylephrine, respectively. These findings are similar to those observed after aerobic exercise training in female-ovariectomized SHR (Sanches et al., 2012; da Palma et al., 2016). However, the decrease in AP and the improvement in baroreflex sensitivity induced by resistance training were significantly less pronounced than in aerobic training in this model (da Palma et al., 2016).

Exercise training also promotes an effective adaptation to vascular autonomic modulation (VAR-SAP and LF-SAP), total HR variance (VAR-PI), cardiac vagal cardiac modulation (RMSSD), and cardiac sympathovagal balance when compared to SHR sedentary animals. These effects are likely to reduce AP and HR after combined exercise training in hypertensive ovariectomized rats. Therefore, we suggest that combined exercise at low/moderate intensity may reduce cardiac and vascular sympathetic modulation, while increasing parasympathetic modulation, and thus improving cardiovascular autonomic balance. Previous studies have shown that aerobic and resistance exercise training improves total HR variance (VAR-PI) and cardiac vagal cardiac modulation (RMSSD) in female-ovariectomized hypertensive rats (da Palma et al., 2016). However, our study is the first to show a reduction in AP associated with improvement in cardiac sympathovagal balance and sympathetic vascular modulation promoted by combined exercise in hypertension with menopause. Since the increase in blood pressure variability is strongly associated with end-organ damage in hypertension (Irigoyen et al., 2016), our findings point to additional benefits of combined exercise training when compared to either aerobic or resistance training alone in the management of hypertension associated with ovarian hormone deprivation.

Our study also show that hypertensive animals have higher TNF levels associated with increased oxidative stress, as shown by lipoperoxidation (TBARS) and protein oxidation in cardiac and renal tissues. These results corroborate previous findings reporting that TNF induces oxidative stress in cardiomyocytes from normotensive rats (Dhingra et al., 2007). On the other hand, experimental data have demonstrated higher NADPH oxidase activity associated with the production of reactive oxygen species; and the sympathetic nervous system seems to have an important role in this relationship (Corbi et al., 2013). Previous studies have shown that AP activates NADPH oxidase and increases oxidative stress in male SHR (Masson et al., 2014). In line with these, our findings show higher renal NADPH oxidase levels in hypertensive animals. It should be stressed that our findings demonstrate that surgical ovariectomy increases renal NADPH oxidase activity, cardiac and renal TBARS and carbonyls, which may contribute to the higher incidence of hypertension in menopausal women.

Moreover, the imbalance between oxidation and antioxidation must be taken into account. Our results show that hypertensive animals also have higher protein oxidation levels (in heart and kidneys) and decreased cardiac catalase. We observed that combined exercise reduces TNF and IL-6 levels in the heart and kidneys, while increasing IL-10 levels in kidneys. Combined exercise training reduces oxidative stress damage in the heart and kidneys, promoted by hypertension and ovariectomy, as shown by the reduction in protein oxidation and lipoperoxidation levels. This beneficial response to chronic combined exercise may be mediated by several mechanisms including a reduction in NADPH oxidase, and an increase in enzymatic (CAT and SOD) or non-enzymatic antioxidants (TRAP). Previous studies undertaken by our group have found that moderate intensity aerobic or resistance exercise training can also reduce cardiac oxidative stress in female-ovariectomized hypertensive rats (da Palma et al., 2016). However, the findings here presented shed new light on the benefits of combined exercise training in modulating inflammation in cardiac and renal tissue, along with oxidative stress in kidneys.

In this study, combined exercise training reduced body weight in ovariectomized animals, thus suggesting a potential clinical implication for menopausal women. We also observed that exercise improves physical capacity as evaluated by their response to the maximal speed and load tests and observed in other studies involving SHR undergoing aerobic or resistance training (Bertagnolli et al., 2006; Sanches et al., 2012; Grans et al., 2014; Sanches et al., 2014). The effectiveness of our exercise training protocol is further supported by the improvement in resting bradycardia of the ovariectomized animals.

Some limitations of this study should be mentioned. First, the lack of ovariectomized sedentary and trained control groups. Second, we did not include aerobic training or resistance training groups. However, those groups have been already studied in previously published manuscripts of our group (Irigoyen et al., 2005; Flues et al., 2010; da Palma et al., 2016). Third, the food consumption was not measured in this experiment. Another limitation of this study may lie in the analysis of oxidative stress and inflammatory profile, which was restricted to some hypertension-target organs, such as cardiac and renal tissues, and did not include other organs, such as muscles and serum, which are also implicated in exercise training induced-neuroimmune modulation.

It is worth mentioning that neural networks are effective mechanisms selected by evolution to coordinate physiological homeostasis. In this sense, studies have shown that autonomic nervous system can modulate inflammation, and consequently, oxidative stress (Pavlov and Tracey, 2012). Therefore, we reasoned that combined exercise training-induced increase in baroreflex sensitivity may reduce inflammatory cytokines both locally and systemically, which may lead to improved redox balance in hypertension target organs, thus reducing AP. In conclusion, combined exercise reduces AP, associated with benefits for baroreflex sensitivity and cardiovascular autonomic modulation, while decreasing inflammation and oxidative stress in hypertensive ovariectomized rats. These findings suggest a positive role of combined exercise training in the management of cardiovascular risk in hypertension associated with ovarian hormone deprivation.

## Author Contributions

GS, DS, and CM conducted most of the experiments. SL contributed to oxidative stress analysis. IS carried out the tissue distribution analysis. LU, M-CI, and KDA designed the project. GS and IS participated in data analysis. GS, LU, and KDA wrote the manuscript and prepared the figures.

## Conflict of Interest Statement

The authors declare that the research was conducted in the absence of any commercial or financial relationships that could be construed as a potential conflict of interest.
